# Bright Blue Emitting Cu-Doped Cs_2_ZnCl_4_ Colloidal Nanocrystals

**DOI:** 10.1021/acs.chemmater.0c02017

**Published:** 2020-06-05

**Authors:** Dongxu Zhu, Matteo L. Zaffalon, Valerio Pinchetti, Rosaria Brescia, Fabrizio Moro, Mauro Fasoli, Marco Fanciulli, Aiwei Tang, Ivan Infante, Luca De Trizio, Sergio Brovelli, Liberato Manna

**Affiliations:** † Department of Chemistry, School of Science, Beijing JiaoTong University, Beijing 100044, China; ‡ Nanochemistry Department, Istituto Italiano di Tecnologia, Via Morego 30, 16163 Genova, Italy; § Dipartimento di Scienza dei Materiali, Universitá degli Studi di Milano-Bicocca, via R. Cozzi 55, 20125 Milano, Italy; ∥ Electron Microscopy Facility, Istituto Italiano di Tecnologia, Via Morego 30, 16163 Genova, Italy; ⊥ Department of Theoretical Chemistry, Vrije Universiteit Amsterdam, De Boelelaan 1083, Amsterdam, 1081 HV The Netherlands

## Abstract

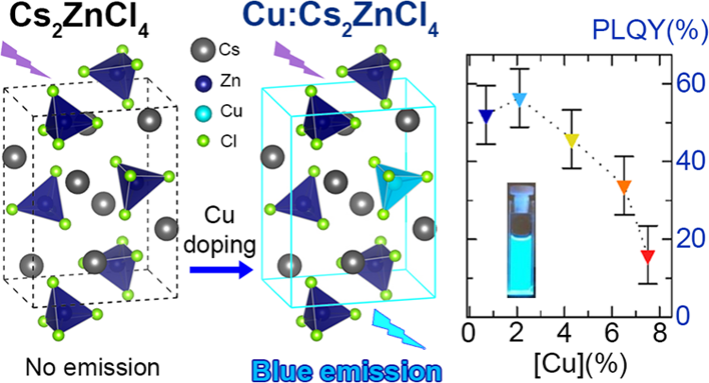

We report here the synthesis of undoped and Cu-doped Cs_2_ZnCl_4_ nanocrystals (NCs) in which we could tune the concentration of Cu from 0.7 to 7.5%. Cs_2_ZnCl_4_ has a wide band gap (4.8 eV), and its crystal structure is composed of isolated ZnCl_4_ tetrahedra surrounded by Cs^+^ cations. According to our electron paramagnetic resonance analysis, in 0.7 and 2.1% Cu-doped NCs the Cu ions were present in the +1 oxidation state only, while in NCs at higher Cu concentrations we could detect Cu(II) ions (isovalently substituting the Zn(II) ions). The undoped Cs_2_ZnCl_4_ NCs were non emissive, while the Cu-doped samples had a bright intragap photoluminescence (PL) at ∼2.6 eV mediated by band-edge absorption. Interestingly, the PL quantum yield was maximum (∼55%) for the samples with a low Cu concentration ([Cu] ≤ 2.1%), and it systematically decreased when further increasing the concentration of Cu, reaching 15% for the NCs with the highest doping level ([Cu] = 7.5%). The same (∼2.55 eV) emission band was detected under X-ray excitation. Our density functional theory calculations indicated that the PL emission could be ascribed only to Cu(I) ions: these ions promote the formation of trapped excitons, through which an efficient emission takes place. Overall, these Cu-doped Cs_2_ZnCl_4_ NCs, with their high photo- and radio-luminescence emission in the blue spectral region that is free from reabsorption, are particularly suitable for applications in ionizing radiation detection.

## Introduction

**Scheme 1:**
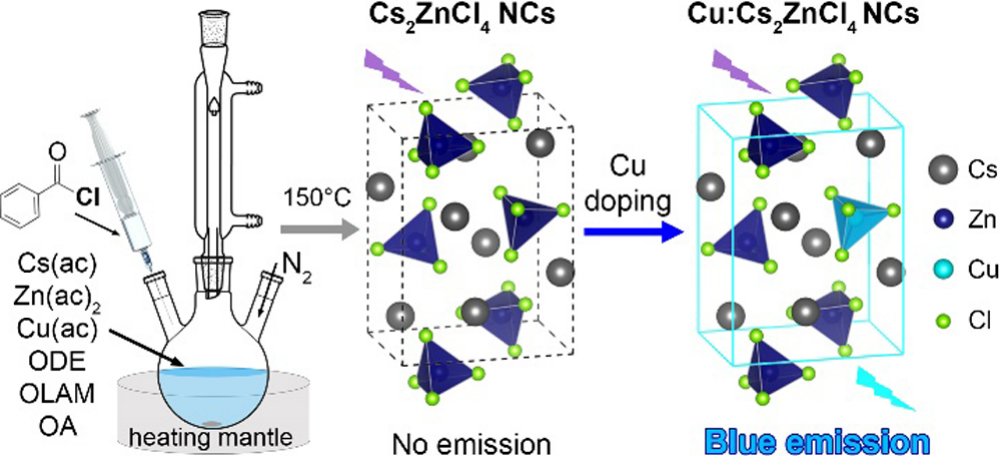
Synthesis of Cs_2_ZnCl_4_ and Cu-Doped Cs_2_ZnCl_4_ NCs

Cs_2_ZnCl_4_ is a wide band gap material of interest for scintillators (high-energy X-ray detection) because it features high detection efficiency and timing resolution, thanks to its fast Auger-free luminescence with emission in the ultraviolet spectral region (4.2 eV).^1,2^ Cs_2_ZnCl_4_ has an orthorhombic crystal structure, with disconnected ZnCl_4_^2-^ tetrahedra that are charge balanced by Cs^+^ cations (Scheme 1), which fill the voids between the tetrahedra.^2−4^ Cs_2_ZnCl_4_ has also been used as a host for Ce^3+^ ions (with a 20% increase of the scintillation light yield)^3^ and for Mn^2+^, Cu^2+^, and Ni^2+^ ions (as substitutional dopants) to study d–d transitions in transition metal M(II) ions in a tetrahedral coordination.^5−7^ Recent studies on the emission properties of Mn(II) ions in tetrahedral coordination have revealed potential interest in Mn-doped hybrid organic–inorganic and fully inorganic zinc(II) halide bulk powders, which also include Cs_2_ZnCl_4_, as green emitters.^8,9^ Yet, to date, the optical properties of Cs_2_ZnCl_4_ on the nanoscale have not been investigated, nor has any potential doping been attempted, although doping of metal halide NCs has been demonstrated to be an effective way to boost the optical properties of these compounds.^10−14^ This work aims at addressing this aspect and is also motivated by the quest for finding nontoxic metal halide NCs with optical properties comparable to those of APbX_3_ perovskite (A = Cs^+^, CH_3_NH_3_^+^, CH(NH_2_)_2_^+^; X = Cl, Br, I) NCs.^12,15−17^

In this work, we have developed a colloidal synthesis of undoped and Cu-doped Cs_2_ZnCl_4_ NCs (with control on the concentration of Cu dopants) and have examined their optical properties. We find that Cs_2_ZnCl_4_ NCs had a band gap of 4.8 eV with no detectable photoluminescence (PL); the Cu-doped samples instead were characterized by a bright blue emission at ∼2.6 eV, mediated mostly by band-edge absorption; according to density functional theory (DFT) calculations, the origin of the blue emission is ascribed to Cu(I) ions, embedded as [CuCl_3_]^2–^ units, which generate localized intragap states that lead to emission via trapped excitons. Interestingly, we observed radio-luminescence (RL) emission at the same energy (∼2.55 eV) when exciting the Cu-doped Cs_2_ZnCl_4_ NCs with X-rays. This makes such NCs particularly interesting for ionizing radiation detection, where such emission can easily be coupled to photomultiplier tubes (which are particularly responsive in the blue spectral region) and to Si detectors.^18,19^

## Results and Discussion

**Figure 1:**
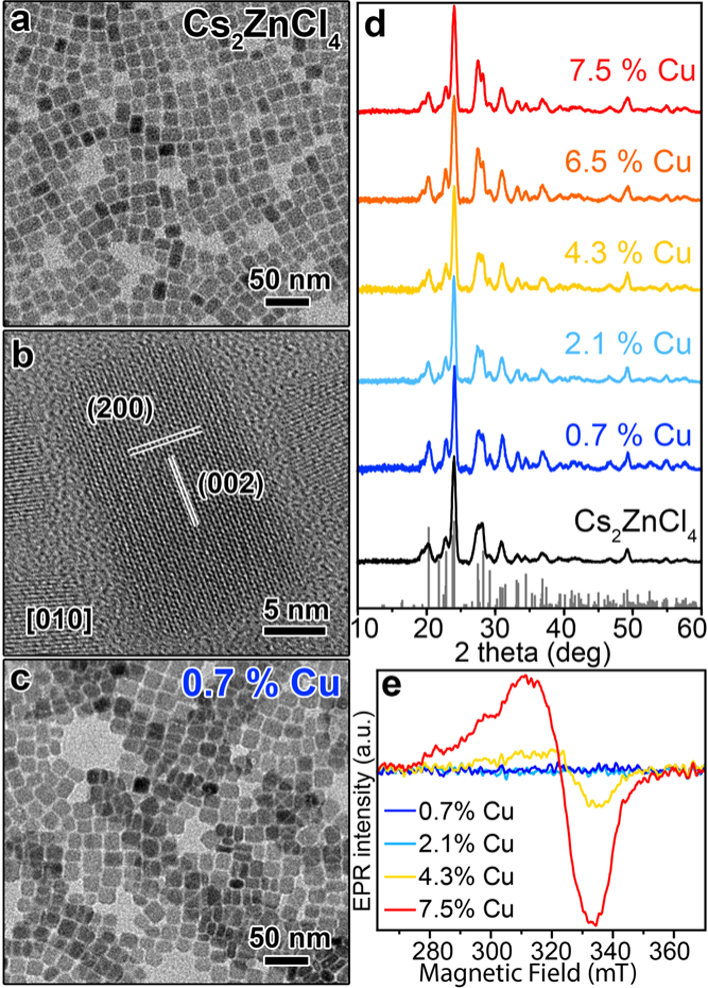
TEM images of (a) Cs_2_ZnCl_4_ and(c) 0.7% Cu-doped Cs_2_ZnCl_4_ NCs. (b) HRTEM image of a Cs_2_ZnCl_4_ NC. (d) XRD patterns of Cs_2_ZnCl_4_ and Cu-doped Cs_2_ZnCl_4_ NC samples with the corresponding bulk reflections (gray bars) of the orthorhombic Cs_2_ZnCl_4_ crystal structure (ICSD number 6062). (e) EPR powder spectra of randomly oriented Cu-doped Cs_2_ZnCl_4_ NC samples, having Cu contents of 0.7, 2.1, 4.3, and 7.5%, respectively, recorded at room temperature and after background subtraction and smoothing.

NCs were synthesized by an approach that is similar to the one followed for halide perovskite NCs,^12,20^ using octadecene, oleylamine, and oleic acid as surfactants, metal acetates as metal precursors, and benzoyl chloride as a precursor for chloride ions (Scheme 1). By varying the Cu/Zn precursor ratio, we could prepare Cu-doped Cs_2_ZnCl_4_ NC samples with a Cu amount ranging from 0.7 to 7.5% (at % with respect to Zn), as measured by energy-dispersive X-ray spectroscopy in a scanning electron microscope and by inductively coupled plasma optical emission spectroscopy (Table S1 and Figure S1 of the Supporting Information (SI)). All samples were composed of NCs having a parallelepiped shape, with a mean size around 17 nm (Figure 1a,c and Figure S2), and an orthorhombic Pnma Cs_2_ZnCl_4_ crystal structure (ICSD number 6062), as revealed by bright-field transmission electron microscopy (TEM) and X-ray powder diffraction (XRD), respectively (Figure 1d).

In none of the Cu-doped NC samples, we found traces of secondary phases (Figure 1d). High-resolution TEM (HRTEM) indicated that all products were composed of monocrystalline particles, with a structure matching the orthorhombic Cs_2_ZnCl_4_ phase (Figure 1b and Figure S3), in agreement with the XRD data. To assess the oxidation state of Cu and Zn in the NCs, we first employed X-ray photoelectron spectroscopy (XPS), which however did not provide reliable results as the NCs degraded quickly under X-ray irradiation. We then performed electron paramagnetic resonance (EPR) spectroscopy on undoped Cs_2_ZnCl_4_ NCs and on four representative Cu-doped Cs_2_ZnCl_4_ NC samples, containing 0.7, 2.1, 4.3, and 7.5% of Cu, respectively, to probe the presence of paramagnetic Cu(II) species (Figure 1e).^21^ The EPR spectra of the undoped and of both the 0.7 and 2.1% Cu-doped Cs_2_ZnCl_4_ NCs showed no EPR signal, indicating the absence of Cu(II) species and therefore suggesting the presence of diamagnetic Cu(I) ions (Figure 1e). On the other hand, the EPR spectra of the 4.3 and 7.5% Cu-doped Cs_2_ZnCl_4_ NC samples were characterized by a signal that was ascribed to Cu(II) species, as corroborated by the simulation of the low temperature (T = 15 K) EPR measurements (Figure S4). The simulated EPR spectrum of the 4.3% Cu-doped Cs_2_ZnCl_4_ NC sample was compatible with the magnetic resonance transitions of Cu(II) ions (S = 1/2) and, specifically, with the interactions of their d-electrons with the nuclear spins of the ^63,65^Cu (*I* = 3/2) and the surrounding ^35,37^Cl isotopes (*I* = 3/2, *n.a.* = 75.78%), (Figure S4).^22−24^ Moreover, EPR indicated that Cu(II) adopted an axial symmetry within the distorted tetrahedrally coordinated Cl^–^ anions (i.e., occupying Zn(II) sites), in the NC crystal structure (see also the computational section below and Figure S4 in the Supporting Information for details).^22^

Overall, these results indicated that doping of Cs_2_ZnCl_4_ NCs proceeds via the introduction of Cu(I) ions up to a Cu concentration of 2.1%, above which Cu cations in both +1 and +2 oxidation states were introduced. Since our syntheses were performed under an inert atmosphere using a Cu(I) precursor, the formation of Cu(II) cations was tentatively ascribed to the disproportionation of Cu(I) ions. The presence of Cu(II) cations as dopants in Cs_2_ZnCl_4_ is not surprising, given the existence of the Cs_2_CuCl_4_ compound, isostructural with Cs_2_ZnCl_4_ (in which Cu(II) ions are pseudotetrahedrally coordinated to Cl^–^ anions) and also recalling that Cu(II) doping of bulk Cs_2_ZnCl_4_ crystals has been already reported.^5,7,25^ On the other hand, the introduction of Cu(I) ions as dopants in Cs_2_ZnCl_4_ is less obvious and had not been reported to date.

**Figure 2:**
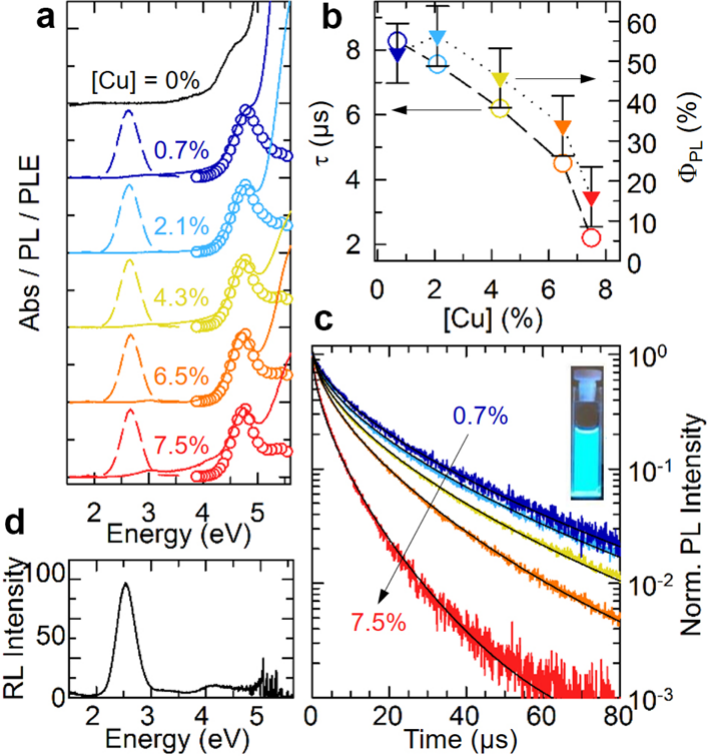
(a) Optical absorption (filled lines), PL (dashed lines) and PLE (empty circles) spectra of undoped and Cu-doped Cs_2_ZnCl_4_ NCs at different Cu concentrations. The spectra are shifted vertically for clarity. (b) PL quantum yields (Φ_PL_, filled triangles) and effective decay lifetime (τ) calculated from the data in (c) as extracted by the fitting procedure with a stretched exponential decay (open circles) as a function of [Cu]. (c) Time-resolved PL decay traces collected at the PL maximum normalized to the intensity of 0.7% Cu-doped NCs. The black lines are the results of the fitting procedure of the experimental decay curves with a stretched exponential decay function. Inset: photograph of a sample illuminated with 4.9 eV light. (d) RL spectrum of 2.1% Cu-doped Cs_2_ZnCl_4_ NCs exposed to X-rays.

We then investigated the optical properties of undoped and Cu-doped Cs_2_ZnCl_4_ NCs (Figure 2). The absorption spectra of all the samples featured a main peak at ∼4.8 eV for all systems, indicating that the introduction of Cu did not modify the main electronic transitions at the band edge (Figure 2a). In the Cu-doped NC samples, there was an additional weak absorption peak at ∼3.02 eV (Figure S5), which we ascribed to localized states of Cu (I) ions (see below). Notably, while the undoped NCs were not emissive, the addition of Cu led to a bright blue PL at ∼2.6 eV (full width at half maximum, FWHM, of 0.45 eV, Figure S6) that was Stokes-shifted from the absorption peak by ∼2.15 eV (Figure S7) and by 0.37 eV with respect to the weak absorption at 3.02 eV. These optical properties were quite different from those of the recently reported Cu-based compounds, namely, CsCu_2_Cl_3_ and Cs_3_Cu_2_Cl_5_, which are characterized by a PL emission at about 2.35 eV. We therefore believe that the local structure surrounding the Cu(I) ions in our system is different from that of Cu(I) ions in those materials.^26,27^ To check whether the emission of our NCs was due to our specific reaction protocol, we tested an alternative colloidal synthesis approach in which Cs-oleate was hot-injected into a solution of CuCl and ZnCl_2_ dissolved in octadecene, oleylamine, and oleic acid, to trigger the formation of Cu-doped Cs_2_ZnCl_4_ NCs. The product NCs featured optical properties that are analogue to those of the Cu-doped NCs reported here, albeit with a much lower PL emission intensity, thus confirming our findings (Figure S8). The PL excitation (PLE) spectra collected at the emission maximum matched closely with the features of the absorption curves: (i) a very weak PLE peak ∼3.02 eV (see Figure S9) and (ii) a main peak at ∼4.8 eV, indicating that the intragap PL was mostly mediated by band-edge absorption. Both the PL and the respective PLE peak positions were independent on the doping level. In turn, the Cu content had significant impact on the emission quantum yield (Φ_PL_) that had its maximum value (∼57 ± 7%) for a Cu concentration of 2.1% and then decreased monotonically to 15 ± 5% for the highest doping level explored (7.5% Cu, Figure 2b). Consistently, time-resolved PL revealed a gradual acceleration of the decay kinetics with the doping level (Figure 2c) that occurred with no measurable modification of the zero-delay PL intensity.

The PL decay curves were well modeled with a stretched exponential function *I*(t) = *I*_0_ × exp ( – (t/τ)^β^) that describes the luminescence dynamics of systems featuring a distribution of decay rates due to local structural disorder, as it likely occurs in our NCs due to the different local environments of trapped exciton states.^28,29^ The obtained trend of PL lifetimes (τ) as a function of the Cu concentration followed that of the Φ_PL_ (Figure 2b). Also, the fitting of the PL decay curves yielded a nearly constant stretching factor (β) (Figure S10), suggesting that the local disorder is essentially independent of the Cu content. This is consistent with the constant PL spectral linewidth of all investigated NCs (Figure S6). The good match between the Φ_PL_ and PL lifetime trends suggests that the weakening of the luminescence with increasing the Cu doping level results from the activation of a nonradiative decay pathway that competes with the radiative recombination of trapped excitons on a similar timescale. In turn, the independence of the early-times PL intensity on doping indicates that trapping in the Cu(I) centers occurs faster than both radiative and nonradiative processes in any investigated sample. Since both the stretching factor β and the FWHM of the PL spectra are essentially independent of the Cu content, an increase in the local disorder cannot be accounted for the observed PL quenching. We thus speculate that the PL losses might arise from concentration quenching processes in the presence of Cu(II) sites that act as PL killer centers. This hypothesis was corroborated by a control experiment in which we exposed the 2.1% Cu-doped NCs to air: we observed that while the NCs preserved their structural integrity, their PL emission gradually decreased with time, most likely due to the partial oxidation of Cu(I) species to nonemissive Cu(II) centers (Figure S11).

The luminescence under ionizing radiation was evaluated by means of RL measurements.^30^ The 2.1% Cu-doped Cs_2_ZnCl_4_ NCs were deposited on a steel disc and exposed to X-rays. The main RL emission band for the NCs was observed at ∼2.55 eV (FWHM 0.458 eV) similar to the one detected in PL (Figure 2d). A further weak emission was detected around 4.2 eV, in analogy to what was reported for the bulk Cs_2_ZnCl_4_ material.^1,2^ Considering the blue RL emission (which pairs well to the peak efficiency of photodetectors typically employed in scintillator devices), a decay time in the order of μs and a substantial suppression of reabsorption as a result of a very large Stokes shift render these Cu-doped NCs as important candidates for ionizing radiation detection applications such as X-ray screens or high-density scintillation systems.^18,19^

**Figure 3:**
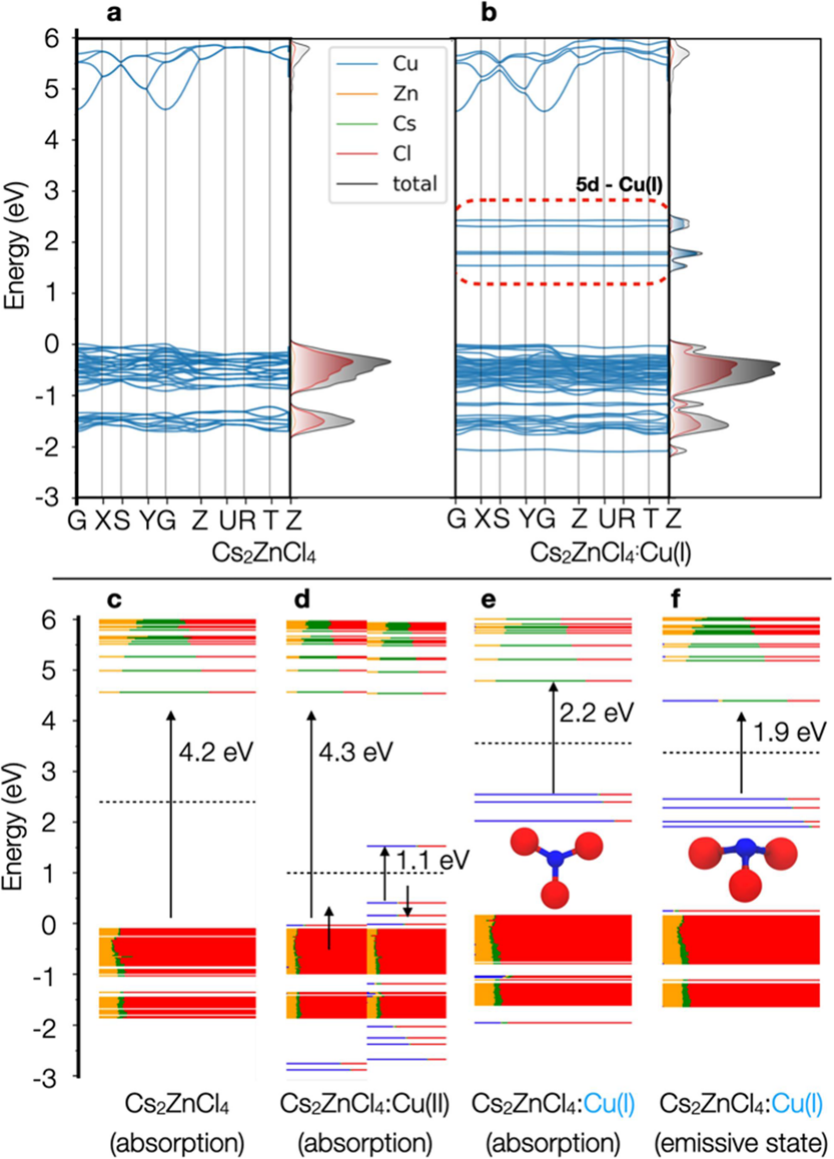
Band structure of (a) pure Cs_2_ZnCl_4_ and (b) Cs_2_ZnCl_4_:Cu(I), where one Zn(II) ion was replaced by a Cu(I) ion. Calculations were carried on a 1 × 1 × 1 orthorhombic unit cell belonging to the Pnma space group and Bravais lattice symbol oP1. Electronic structure at the Γ point of a 2 × 2 × 2 unit cell computed for (c) Cs_2_ZnCl_4_, (d) Cs_2_ZnCl_4_:Cu(II), and (e) Cs_2_ZnCl_4_:Cu(I). In (e), the reduced Cu(I) was obtained by removing one Cl ion directly attached to Cu, thus formally switching from a tetrahedral [CuCl_4_]^2–^ to a trigonal [CuCl_3_]^2–^ unit. Cell parameters and ionic positions of all systems were relaxed in the ground state. The electronic structure of the reduced system in (f) has been obtained by relaxing cell parameters and ionic position in the triplet state followed by a single-point calculation in the singlet state at this new geometry. This provides a hint on the electronic structure of the emissive state. All calculations were carried out at the DFT/PBE level of theory.

To unravel the origin of the PL emission from the Cu-doped NCs, we carried out DFT calculations using the cp2k 6.1 quantum chemistry software package.^31^ First, we computed the band structure of the undoped Cs_2_ZnCl_4_ system (Figure 3a). The presence of disconnected tetrahedral units suggests that the band gap remains unchanged, moving from the bulk to the nanoscale system. At the DFT/PBE level of theory,^32^ on a 1 × 1 × 1 cell, after cell and atomic position relaxation, we found a direct 4.5 eV band gap located at the Γ point. By expanding the cell to 2 × 2 × 2, the gap slightly shrank to 4.2 eV (Figure 3c). This value was lower than the one experimentally observed (4.8 eV, Figure 2a). In the one-electron picture of DFT, the HOMO-LUMO energy difference corresponds to the lowest excited state. However, the pure exchange-correlation (xc) functional, in our case PBE, tends to overly localize the hole, leading to an overestimation of the electron–hole interaction and thus a reduction of the energy gap.^33^ Usually, for solid-state systems, this translates in a huge underestimation of the band gap. On the other hand, the localized nature of 0D systems, visible also from a flat structure of the valence band region in Figure 3a, makes the DFT/PBE approach perform better than the commonly employed range-separated hybrid HSE06 functional for solids (which provides an overestimated gap of 6.1 eV for a 1 × 1 × 1 cell for Cs_2_ZnCl_4_). Considering that PBE is also computationally cheaper, we decided to perform the rest of the calculations with this functional, with an educated guess of a constant underestimation of ∼0.6 eV against the experiments for the whole set of systems studied here.

The band structure of Cs_2_ZnCl_4_ had two interesting features: (i) rather localized holes are present, which are visible from the flat structure of the valence band region; (ii) unlike other 0D systems, such as Cs_4_PbBr_6_,^34^ the conduction band is manifestly dispersive, with a marked delocalization of the wavefunction over the 6s orbitals of Cs and the 4s orbitals of Zn, as is evinced in the density of states plotted in Figure 3a,c, the latter computed at the Γ point. We then substituted one Zn ion with a Cu(II) ion in a 2 × 2 × 2 cell, which corresponds to a Cu dopant concentration of ∼3%. Notably, the d^9^ configuration of Cu(II) in the local CuCl_4_ unit with *T*_d_ symmetry causes an unequally distribution of the occupied triply degenerate T_2_ orbitals. As a result, a Jahn–Teller distortion occurs, leading to a loss of symmetry and a breaking of the orbital degeneracy favoring a pseudotetrahedral configuration with D_2d_ symmetry. This is evidenced in Table S2, where the Cl-Cu-Cl set of angles is split into two clear sets of roughly 100 and 127°, respectively.

The electronic structure of this system presented a doublet spin-magnetization with broken spin alpha and beta configurations, each identified by up and down arrows in Figure 3d. Here, the band gap was given by a forbidden d–d transition within the Cu(II) ion calculated at 1.1 eV (Figure 3d). The first allowed transition lied at 4.3 eV, which should correspond to ∼ 4.9 eV considering the underestimation of the band gap by DFT/PBE (Figure 3d), close to the value of the undoped system (Figures 3c and 2a). These findings suggested that the PL emission of our Cu-doped NCs could not be ascribed to Cu(II) ions, in line with our EPR data, which indicated the absence of Cu(II) ions in the brightest samples (i.e., 0.7 and 2.1% Cu-doped Cs_2_ZnCl_4_ NCs).

To explain the PL mechanism, we computed the band structure of the Cu(I)-doped Cs_2_ZnCl_4_ system, obtained by removing a chloride ion from the CuCl_4_ tetrahedral unit, which translates into a local [CuCl_3_]^2–^ moiety. In this case, the 3d orbitals of Cu moved inside the band gap of the Cs_2_ZnCl_4_ material (Figure 3b). The calculated gap is now 2.2 eV, which we could estimate to be ∼2.8 eV in the experiment. This is in line with the presence of a small peak at 3.02 eV in absorption and PLE spectra, which was, thus, safely ascribed to Cu(I) (Figure 3e). Another feature observed in this system was that the lowest conduction band states are delocalized and composed of the linear combination of empty 4s orbitals of Cu with both the 6s of Cs and 4s of Zn (Figure S12).

To observe the evolution of the emissive state, we performed a structural relaxation of the triplet state, which we use to mimic the behavior of the excited singlet state. The structure of the CuCl_3_ unit transformed from a planar configuration, obtained in the ground state of Cs_2_ZnCl_4_:Cu(I), to a trigonal pyramidal one in the triplet state, indicating a large structural relaxation, in particular, of the dihedral Cl-Cu-Cl-Cl angle, which goes from 3–5° to ∼115° (Table S2). We then computed the electronic structure of the singlet ground state at the geometry of the relaxed triplet, as shown in Figure 3f. The gap in this configuration lied at 1.9 eV, i.e., at about 2.5 eV considering the DFT/PBE underestimation, well in line with the observed PL emission (∼2.6 eV, Figures 3f and 2a). Notably, the lowest state of the conduction band, i.e., the LUMO, was strongly localized on the CuCl_3_ unit, suggesting that the exciton becomes trapped (consistent with the slow decay time observed experimentally) and providing a gateway for an efficient emission.

Finally, as a general extension of the synthesis methods and concepts discussed in this work, we have also synthesized both undoped and Cu-doped Cs_2_ZnBr_4_ NCs. For this halide system as well, we have found that undoped Cs_2_ZnBr_4_ NCs are not emissive and the presence of Cu dopants induced a PL emission in the blue spectral region (∼2.67 eV, see Figure S13).

## Conclusions

In conclusion, we have developed a colloidal synthesis route to prepare monocrystalline Cs_2_ZnCl_4_ NCs and dope them with different concentrations of Cu ions, ranging from 0.7 to 7.5%. The introduction of Cu(I) dopants conferred a bright intragap PL blue emission at ∼2.6 eV to the Cs_2_ZnCl_4_ NCs, which were otherwise non luminescent. The PL quantum yield was maximized (∼55%) for the sample with a Cu content of 2.1%, and it systematically decreased when increasing the amount of dopant. Furthermore, radio-luminesce emission was observed at analogous energies (∼2.55 eV) when exciting the Cu-doped NCs via X-rays. The origin of this emission was ascribed to Cu(I) ions. These ions introduce intra-band gap states onto which photo-excited excitons become trapped and can provide an efficient emission. Our findings suggest that a broad range of metal halide materials, if rationally engineered, for example via doping procedures, may exhibit very interesting optical properties and could be potentially employed in photodetectors and scintillators. Systems having a high RL emission in the blue spectral region and free from reabsorption, such as Cu-doped Cs_2_ZnCl_4_ NCs, are highly desirable for ionizing radiation detection.

## Experimental Section

### Chemicals

Cesium acetate (Cs(ac), 99.99%), zinc acetate (Zn(ac)_2_, 99.99%), copper(I) acetate (Cu(ac), 97%), 1-octadecene (ODE, 90%), oleylamine (OLAM, 98%), oleic acid (OA, 90%), benzoyl chloride (Bz-Cl, 98%), hexane (anhydrous, 95%), and ethyl acetate (99.9%) were purchased from Sigma-Aldrich. All chemicals were used without any further purification.

### Synthesis of Cu-Doped Cs_2_ZnCl_4_ NCs

To produce Cu-doped Cs_2_ZnCl_4_ NCs, we developed a colloidal hot-injection approach. In a typical synthesis, Cs(ac) (0.2 mmol), Zn(ac)_2_ (0.4-x mmol), a desired amount (x mmol) of Cu(ac), 2 mL of ODE, 1 mL of OLAM, and 1 mL of OA were mixed together in a 50 mL 3-necked round-bottom flask and heated up to 130 °C under vacuum for 1 h. The temperature of the resulting transparent reaction solution was increased up to 150 °C under Ar_2_, and afterward, 200 μL of Bz-Cl dispersed in 0.5 mL of degassed ODE was swiftly injected inside the flask. The reaction was quenched after 3 min by using an ice–water bath. Finally, 4 mL of hexane was added to the crude NC solution, which was centrifuged at 4000 rpm for 5 min. The final NC product was cleaned in the following way: the precipitate was redispersed in 2 mL of hexane and centrifuged at 4000 rpm for 5 min to get rid of possible aggregates; the NCs in the supernatant were precipitated by the addition of 2 mL of ethyl acetate and centrifugation at 4000 rpm for 5 min; the final precipitate was dispersed in hexane (1 mL) and stored in a glass vial in a glove box for further use. All the washing procedures were carried out under an inert atmosphere. Different NC samples with different Cu dopant amounts were prepared by varying the Cu/Zn precursor ratio from 0/10, 0.1/9.9, 0.2/9.8, 0.5/9.5, 1/9, 2/8, and 3/7.

### Transmission Electron Microscopy (TEM) Analysis

The samples were prepared by dropping dilute NC solutions onto carbon-coated 200 mesh copper grids for low-resolution TEM or onto ultrathin carbon/holey carbon-coated 400 mesh gold grids for high-resolution (HR) TEM investigation. Low-resolution TEM analyses were performed on a JEOL JEM-1400Plus microscope with a thermionic gun (LaB_6_ crystal) operated at an acceleration voltage of 120 kV. HRTEM characterizations were performed using an image-C_S_-corrected JEOL JEM2200FS microscope operating at 200 kV. Due to the fast beam damage undergone by the NCs, already reported for halide perovskite NCs,^35^ they were exposed to a relatively low dose rate (∼30 electrons/(Å^2^ s), about 50 times lower than the typical dose rate used for HRTEM imaging). HRTEM images were acquired using a direct electron detector (K2 Summit, Gatan), in super-resolution mode. Each image reported in this work is a portion of the 258 × 267 nm frame obtained by summing aligned 20–50 frames obtained by very short exposure (0.1–0.5 s), with a total acquisition time of 10–20 s. The NCs were overall exposed to a total electron dose lower than that during a typical HRTEM experiment.

### X-ray Diffraction (XRD) Characterization

XRD patterns were acquired with a PANanalytical Empyrean X-ray diffractometer equipped with a 1.8 kW Cu Kα ceramic X-ray tube and a PIXcel3D 2 × 2 area detector, operating at 45 kV and 40 mA. Specimens for XRD measurements were prepared by dropping a concentrated NC solution onto a silicon zero-diffraction single crystal substrate. The diffraction patterns were collected under ambient conditions using a parallel beam geometry and the symmetric reflection mode. XRD data analysis was conducted using the HighScore 4.1 software from PANalytical.

### Scanning Electron Microscopy (SEM)

SEM analysis was carried out on a JEOL JSM-7500FA FE-SEM with a cold field-emission gun (FEG). Energy-dispersive X-ray spectroscopy (EDS, Oxford instrument, X-Max, 80 mm^2^) was used to evaluate the elemental ratios. All experiments were done at 8 mm working distance, 15 kV acceleration voltage, and 15 sweep count for each sample.

### Inductively Coupled Plasma (ICP-OES) Elemental Analysis

ICP elemental analysis performed via inductively coupled plasma optical emission spectroscopy (ICP-OES) with an iCAP 6300 DUO ICP-OES spectrometer (ThermoScientific) was used to quantify the Cu-to-Zn ratio. All chemical analyses performed by ICP-OES were affected by a systematic error of about 5%. The samples were dissolved with 1 mL of aqua regia (HCl/HNO_3_ = 3/1(v/v)) overnight.

### Optical Characterization

Absorption spectra from NC films were recorded using a Cary5000 spectrophotometer equipped with an integrating sphere. The samples were prepared by drop-casting a concentrated suspension on a quartz glass slide. The UV–visible absorption spectra were recorded on a Varian Cary 5000 UV–vis–NIR spectrophotometer. The PL and PL excitation (PLE) spectra were measured on a Varian Cary Eclipse spectrophotometer equipped with a 330 nm long pass filter, having a flat transmission around 90–91%, placed between the sample and the detector. The samples were prepared by diluting NC samples in 3 mL of hexane in 1 cm path length quartz cuvettes with airtight screw caps. The sample preparation was performed inside a nitrogen-filled glovebox.

A quadrupled Nd:YAG (266 nm) Q-switched Continuum Minilite laser (10 ns pulse width, 50 mJ/pulse, 1–15 Hz repetition rate) has been used as an excitation source in time-resolved PL measurements, performed on stirred NC solutions to prevent sample damage. Time-resolved PL decays were collected with a Hamamatsu R943–02 GaAs photocathode Ortec 9353 time-correlated single-photon counting unit (time resolution < 1 ns) coupled to an Oriel Instruments Cornerstone 260 monochromator. The PL quantum yields have been calculated using a 0.5 M quinine sulfate solution in H_2_SO_4_ as a relative standard. The PL spectra required to evaluate the quantum yields of the NC solutions have been collected with a Varian Cary Eclipse spectrophotometer and the absorption spectra with a Varian Cary 50 Scan UV–visible spectrophotometer.

### Radio-Luminescence

The 2.1% C-doped Cs_2_ZnCl_4_ NC sample was excited using a Philips 2274 X-ray tube (with a tungsten target) operated at 20 kV and 20 mA. The spectra were collected at room temperature with a Horiba CP140 spectrometer coupled to a Horiba Syncerity CCD detector. The spectra were corrected for the spectral response of the detection system.

### Electron Paramagnetic Resonance

CW-EPR spectra were recorded on a Varian spectrometer with a Bruker super-High Q cavity (ER 4122SHQE) coupled to a He-flow ESR900 cryostat. The measurements were taken with the following experimental parameters. Microwave frequency: 9.4 GHz, microwave power: 2 mW, magnetic field modulation amplitude: 0.2 mT, magnetic field modulation frequency: 100 kHz, time constant: 300 ms, magnetic field step: 0.005 mT, and temperature *T* = 15 and 298 K.

### DFT Calculations

We have carried out atomistic simulations at the density functional theory level using the PBE exchange–correlation functional as implemented in the VASP 6.1 and CP2K 6.1 packages. Band structure calculations were performed using the VASP package. We used a k mesh grid of 6 × 6 × 6 for the Brillouin zone integration. The atomic positions and the lattice parameters were both relaxed until the forces were smaller than 0.001 Hartree/Angstrom. We used a kinetic energy cutoff of 400 eV and 1 × 1 × 1 unit cell. The electronic structure calculations of the cell doped with Cu were computed on a 2 × 2 × 2 cell and at the Gamma point using the cp2k package, which employs a double-ζ basis set plus polarization functions on all atoms. Both cell parameters and ionic positions were relaxed in this case also.

## Supporting Information Available

SEM-EDS maps, TEM and HR-TEM images, EPR and optical data, and molecular orbital plot (PDF)
