# Supplementary material for: Bright Blue Emitting Cu-Doped Cs2ZnCl4 Colloidal Nanocrystals
Source: Chem Mater. 2020 Jun 5;32(13):5897–903. doi: 10.1021/acs.chemmater.0c02017 (PMC8016065; doi:10.1021/acs.chemmater.0c02017)
Supplement: Supplementary file 1 — cm0c02017_si_001.pdf [file cm0c02017_si_001.pdf]

SUPPORTING INFORMATION

Bright Blue Emitting Cu-doped Cs_2ZnCl_4 Colloidal Nanocrystals

Dongxu Zhu[a,b], Matteo Zaffalon[c], Valerio Pinchetti[c], Rosaria Brescia[d], Fabrizio Moro[c], Mauro Fasoli[c], Marco Fanciulli[c], Aiwei Tang[a], Ivan Infante*[b,e], Luca De Trizio*[b], Sergio Brovelli*[c], Liberato Manna*[b]

[a] Department of Chemistry, School of Science, Beijing JiaoTong University, Beijing 100044, China

[b] Nanochemistry Department and [d] Electron Microscopy Facility, Istituto Italiano di Tecnologia, Via Morego 30, 16163 Genova, Italy

[c] Dipartimento di Scienza dei Materiali, Università degli Studi di Milano-Bicocca, via R. Cozzi 55, 20125 Milano, Italy

[e] Department of Theoretical Chemistry, Vrije Universiteit Amsterdam, De Boelelaan 1083, 1081 HV Amsterdam, The Netherlands

Table S1. Composition of Cu-doped Cs_2ZnCl_4 NCs measured by SEM-EDS and ICP-OES instrument. The amount of different elements was expressed relative to that Zn, which was assumed to be 1.

Cu/Zn precursors ratio	SEM-EDS analysis		ICP-OES Cu/Zn (%)
	Composition	Cu/Zn (%)	
0/10	$Cs_{1.96}ZnCl_{4.57}$	/	/
0.1/9.9	$Cs_{1.87}ZnCl_{4.20}$	/	0.7
0.2/9.8	$Cs_{2.00}ZnCl_{4.48}$	/	2.1
0.5/9.5	$Cs_{1.94}ZnCl_{4.60}$	/	2.7
1/9	$Cs_{1.95}ZnCl_{4.57}$	4.0	4.3
2/8	$Cs_{1.90}ZnCl_{4.62}$	6.0	6.5
3/7	$Cs_{1.84}ZnCl_{4.30}$	7.0	7.5

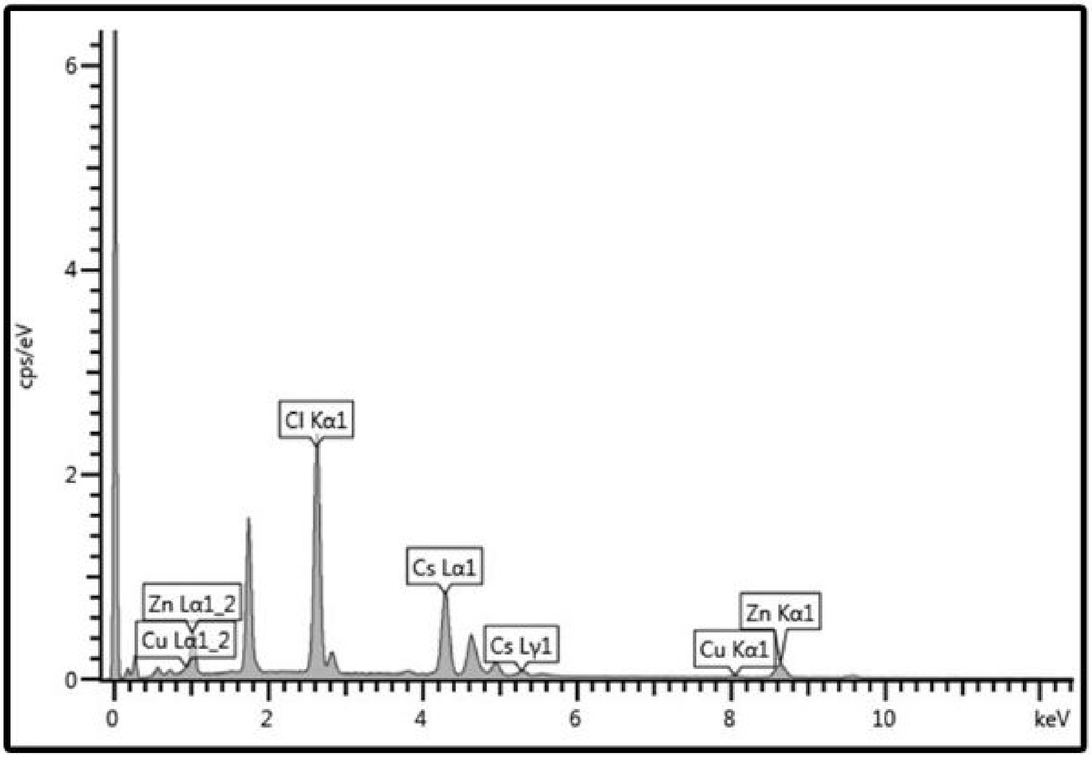

Figure S1. SEM-EDS spectrum collected for the sample with analytical Cu concentration 7.5%.

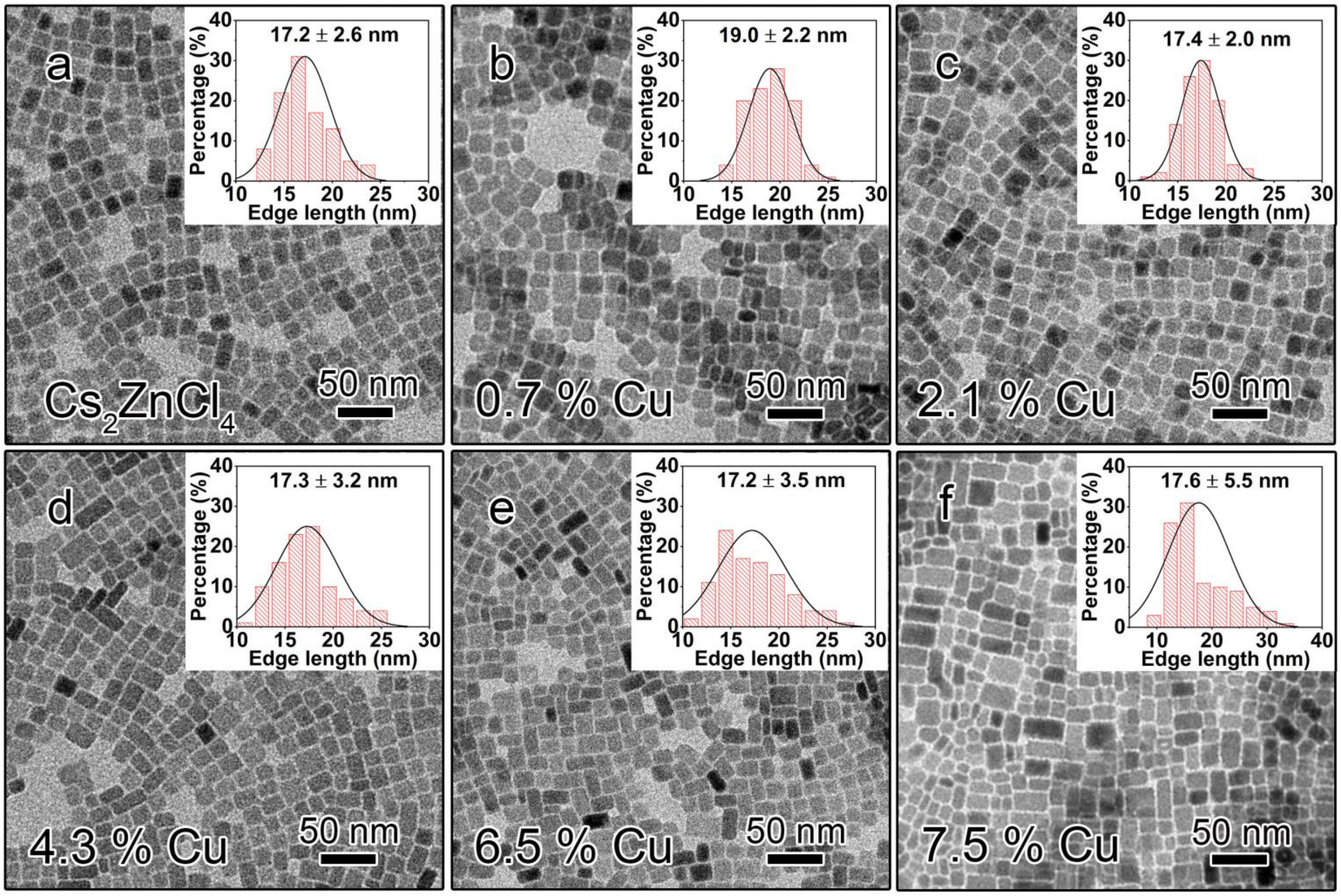

Figure S2. Overview of TEM images with the corresponding size distribution histograms of (a) undoped and Cu-doped Cs_2ZnCl_4 NCs with analytical Cu concentration of (b) 0.7%, (c) 2.1%, (d) 4.3%, (e) 6.5% and (f) 7.5%. The Cu concentration are defined as [Cu]/[Zn] by ICP-OES analysis.

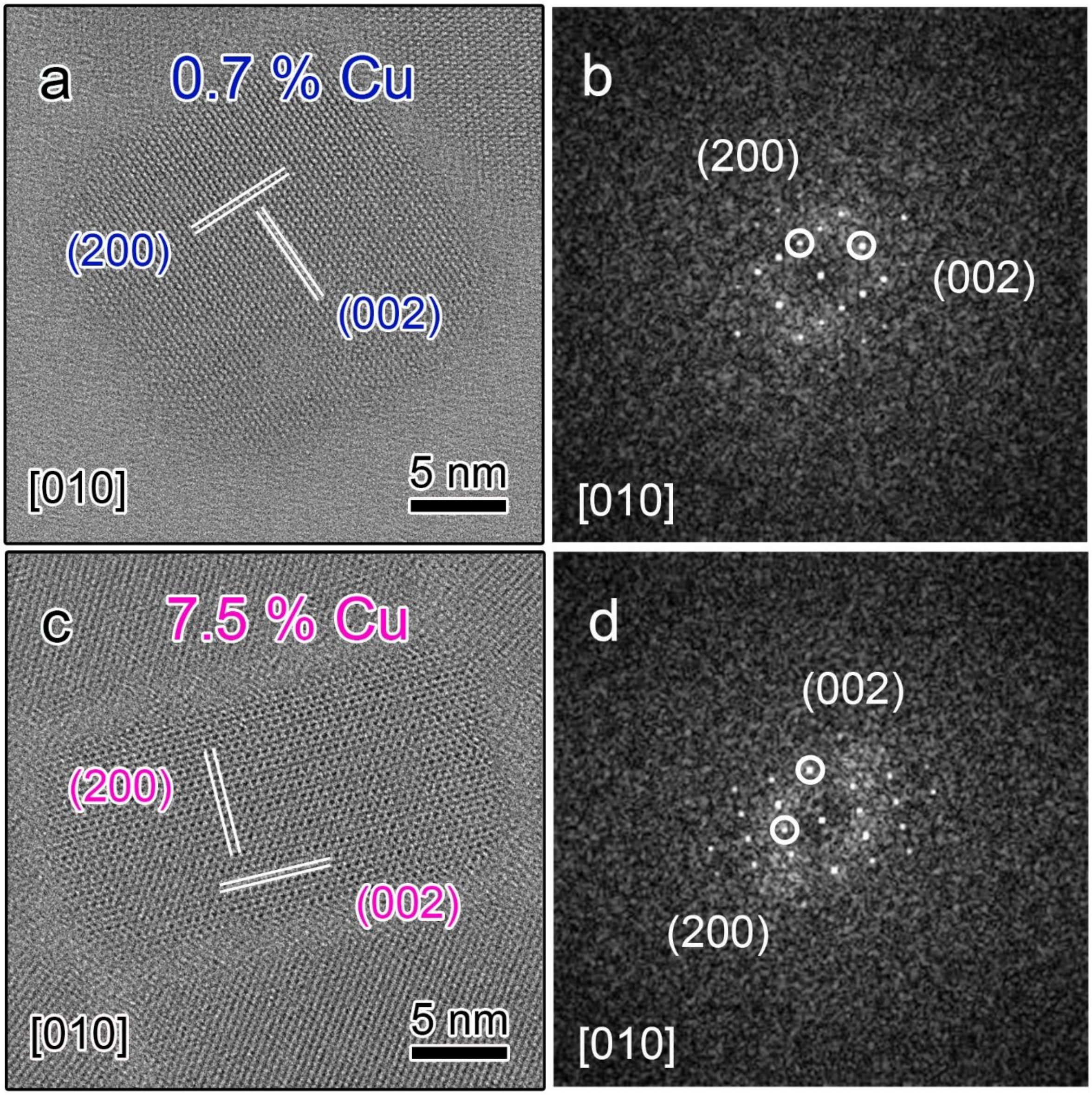

Figure S3. HRTEM image and the corresponding Fast Fourier transforms (FFTs) of one of (a, b) 0.7 % Cu doped Cs_2ZnCl_4 NCs and (c, d) 7.5 % Cu doped Cs_2ZnCl_4 NCs, viewed along their [010] zone-axis.

EPR ANALYSIS

In Figure S4 (right panel) the spectrum acquired for 4.3% Cu-doped Cs_2ZnCl_4 NCs was simulated with the spin-Hamiltonian:

$$\hat{H} = \mu_B \hat{\boldsymbol{B}} \boldsymbol{g} \hat{\boldsymbol{S}} + \hat{\mathbf{S}} \boldsymbol{A}_{\boldsymbol{Cu}} \hat{\mathbf{I}} + \hat{\mathbf{S}} \boldsymbol{A}_{\boldsymbol{Cl}} \hat{\mathbf{I}}$$

where $\boldsymbol{g}$ is the tensor, μ_B is the Bohr magneton, $\hat{\boldsymbol{B}}$ is the magnetic field vector, $\hat{\mathbf{S}}$ and $\hat{\mathbf{I}}$ are the electron and nuclear spin operators respectively, and $\boldsymbol{A}_{\mathbf{Cu}}$ and $\boldsymbol{A}_{\mathbf{Cl}}$ are the hyperfine coupling tensors due to the interactions between the Cu(II) *d*-electron spins and the Cu and Cl nuclear spins respectively.

The simulation provides an axial spectroscopic *g*-factor with parallel and perpendicular values $g_{\perp}$ = 2.089 and $g_{//}$ = 2.45, respectively; and anisotropic hyperfine interactions $A_{Cu,\perp}$ = 119 ± 5 MHz, $A_{Cu,//}$ = 50 ± 5 MHz, $A_{Cl,\perp}$ = 30 ± 5 MHz and $A_{Cl,//}$ = 60 ± 5 MHz.{Merks, et al. *J. Phys. C: Solid State Phys.* **1979,** *12*} The perpendicular and parallel components of the hyperfine tensor $\boldsymbol{A}_{\boldsymbol{Cu}}$ split in 4 lines (*i.e.* 2 I + 1) the EPR peaks centered around 270 mT and 330 mT, respectively; whereas the $\boldsymbol{A}_{\boldsymbol{Cl}}$ tensor provides a further split of the electronic energy levels in 4 lines which are responsible for the linewidth broadening of the EPR peaks.

The linewidth broadening of the EPR spectrum for 7.5% Cu-doped Cs_2ZnCl_4 NCs (left panel) to the Cu(II) - Cu(II) electron spin interactions as result of the decreased averaged distance between the paramagnetic Cu(II) ions.

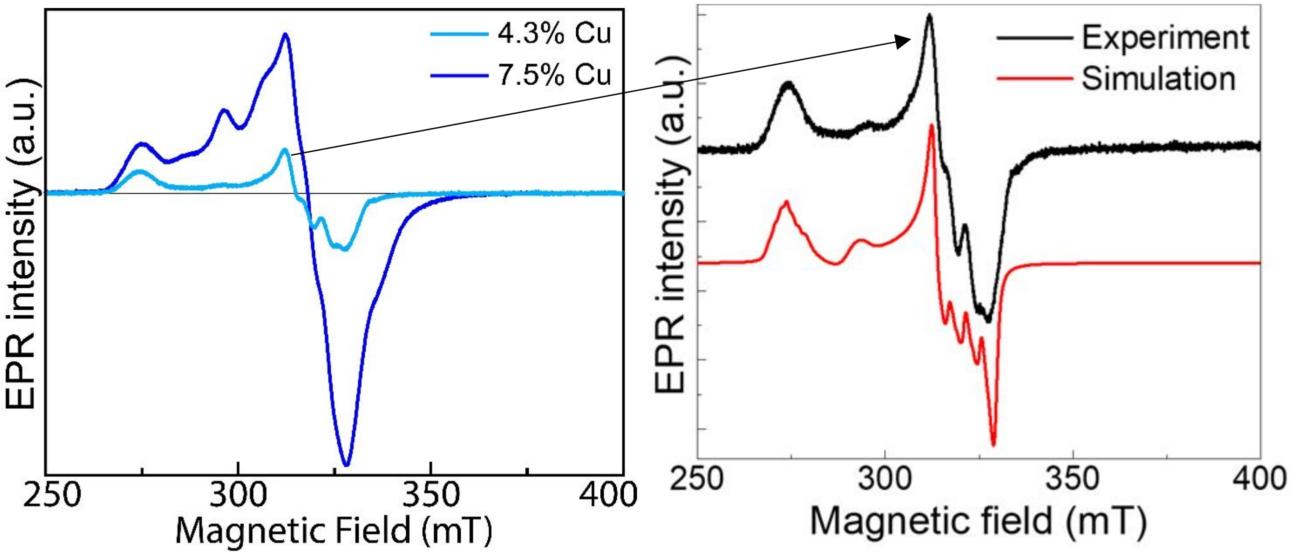

Figure S4. EPR spectra of 4.3% and 7.5% Cu-doped Cs_2ZnCl_4 NCs (left) and the simulation of the EPR spectrum of the 4.3% Cu-doped Cs_2ZnCl_4 NC sample (right).

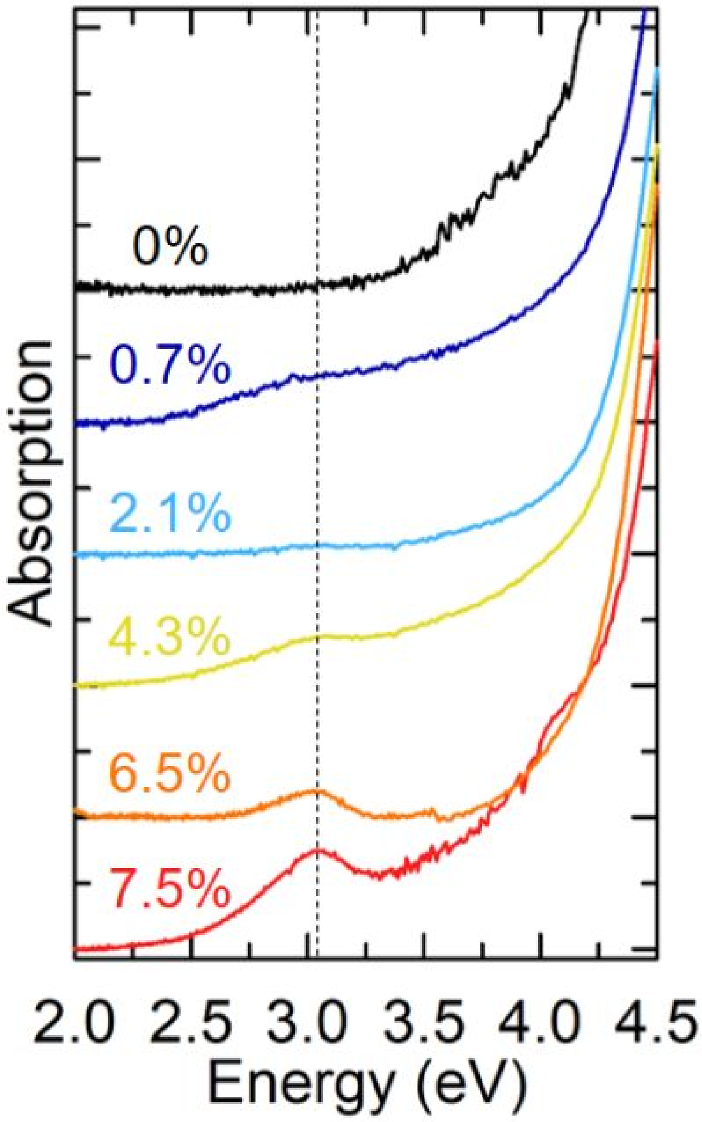

Figure S5. Enlargement of the low-energy portion of the optical absorption spectra of Cu-doped Cs_2ZnCl_4 NCs reported in Figure 2a, highlighting the emergence of an absorption feature at 3.05 eV (dashed vertical line) upon increasing the Cu concentration.

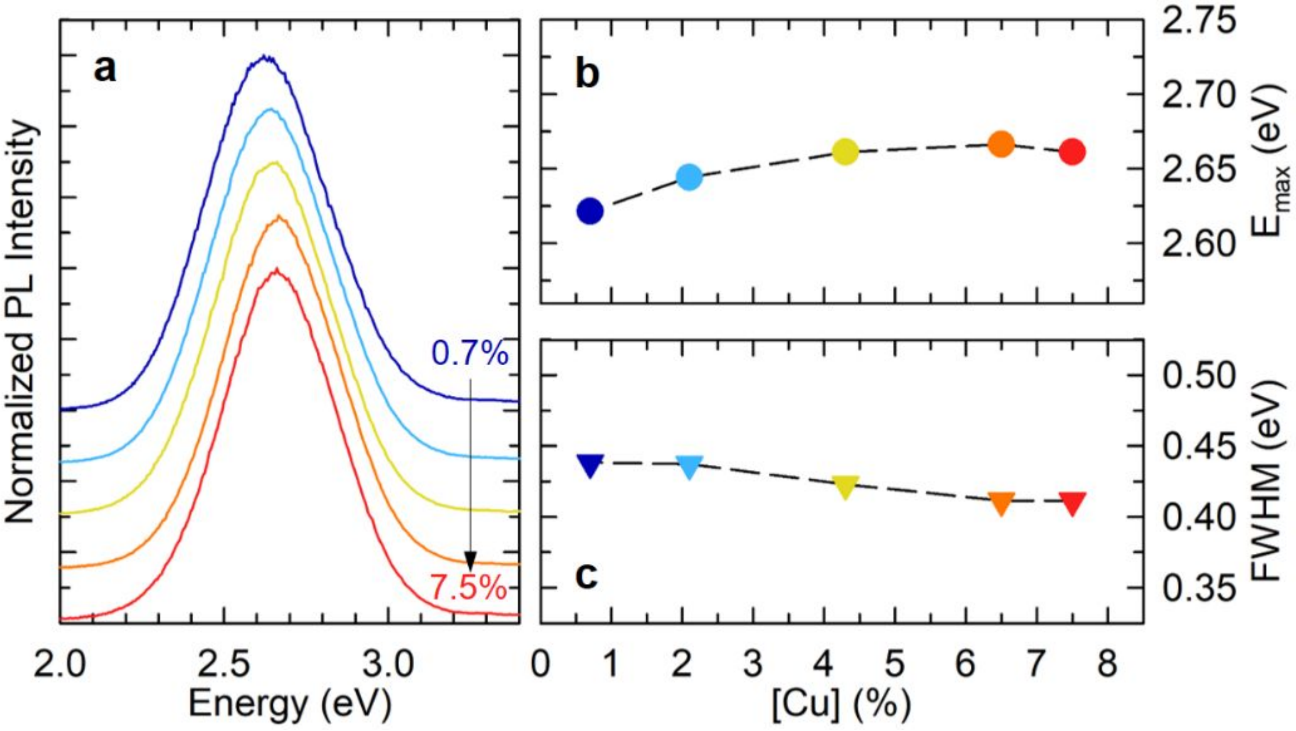

Figure S6. (a) PL spectra of Cu-doped Cs_2ZnCl_4 NCs reported in Figure 2a and the respective Cu concentration dependences of (b) the PL maximum peak energy (E_{max}) and (c) FWHM, both showing nearly invariance upon increasing [Cu].

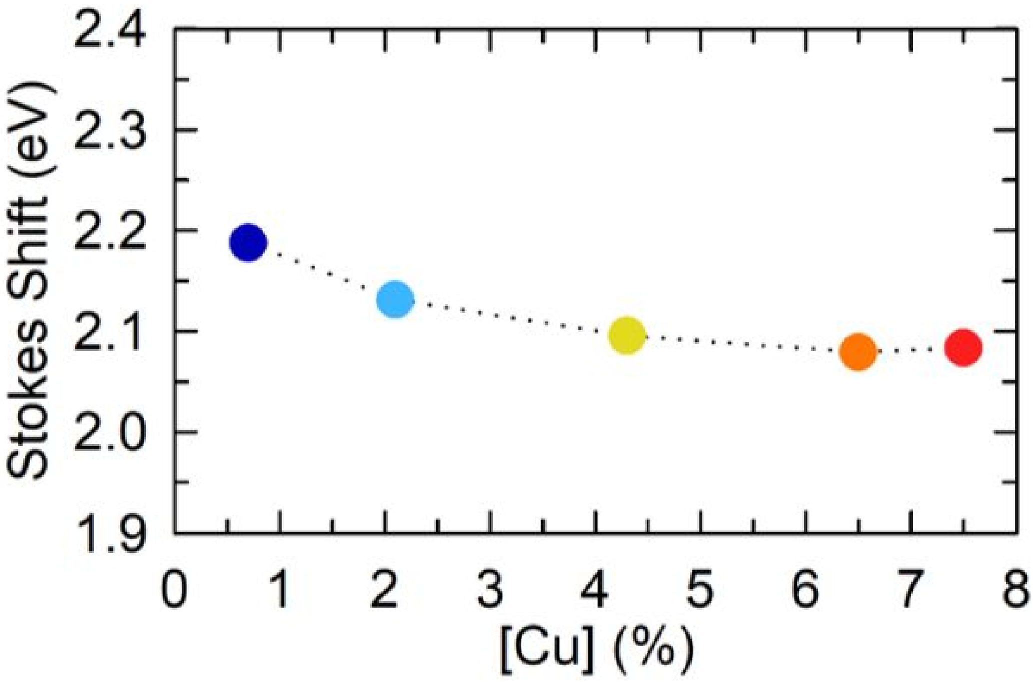

Figure S7. Cu dependence of the Stokes shift of Cu-doped Cs_2ZnCl_4 NCs calculated as the difference between the PLE maximum peak energy and the PL maximum peak energy.

Alternative Synthesis Approach of Cu-doped Cs_2ZnCl_4

We synthesized Cu-doped Cs_2ZnCl_4 NCs following a different colloidal route. A Cs-oleate precursor solution was prepared by dissolving 0.325 g of Cs_2CO_3 with 5 ml of OA on hotplate at 150°C. 0.4 mmol of $ZnCl_2$, 0.08 mmol CuCl, 2 ml ODE, 1 ml OA, 1 ml OLAM were mixed in a 20 ml vial and heated up to 100°C for 30min on a hotplate. Then, the temperature was further increased up to 130°C and 500 µl of the Cs-oleate solution were swiftly injected. After 5min, the reaction was stopped by immersing the vial in a water bath. The TEM images, XRD analysis and optical properties of the obtained NCs are reported in Figure S12.

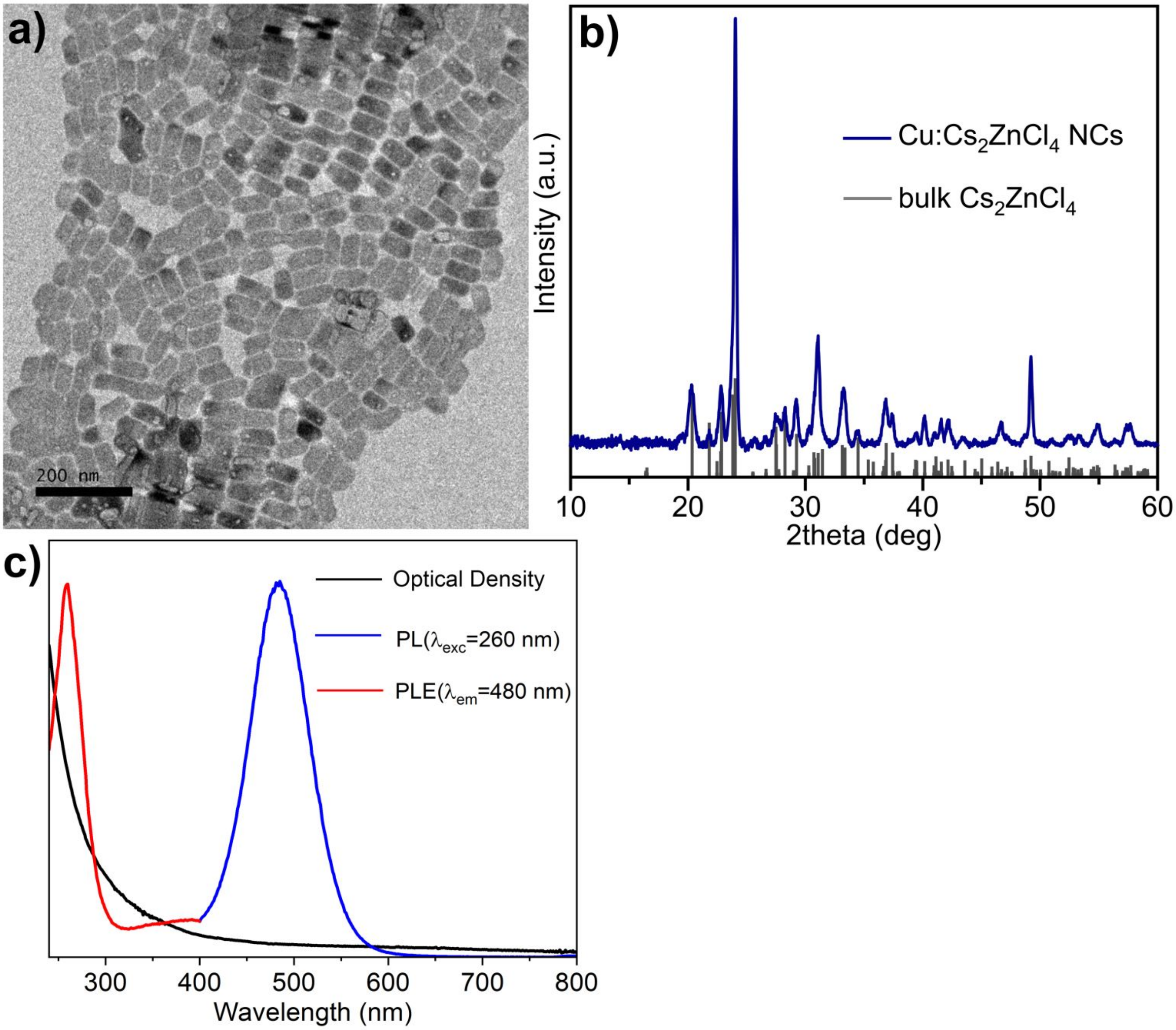

Figure S8. a) TEM micrograph, b) XRD pattern and c) optical properties of Cu-doped Cs_2ZnCl_4 NCs obtained by employing an alternative colloidal approach.

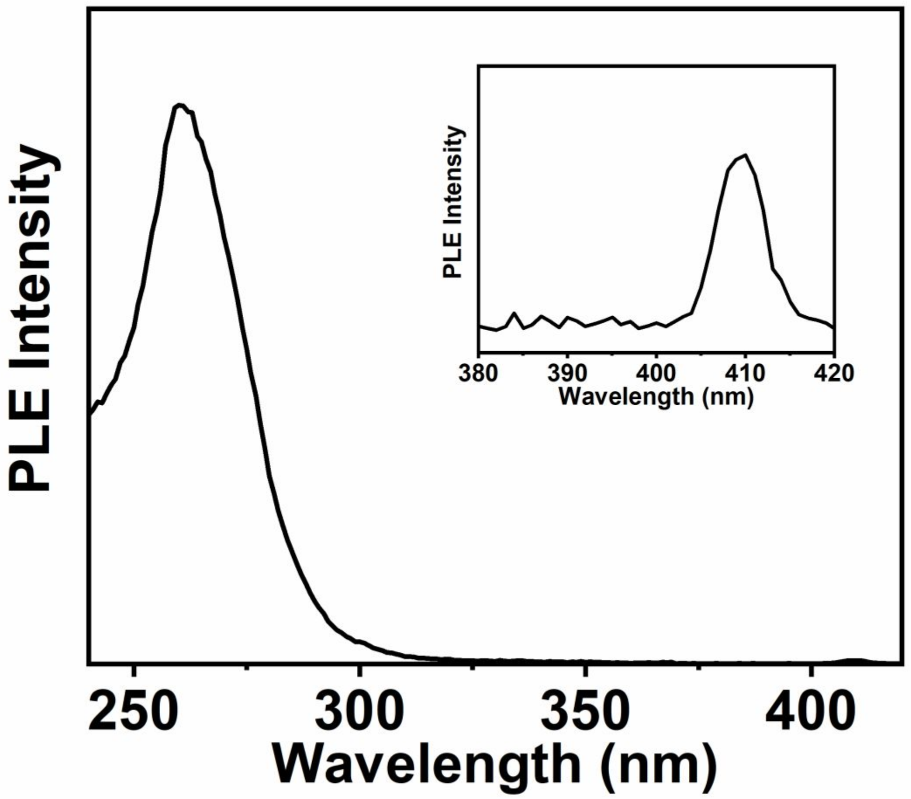

Figure S9. PLE curve of the 0.7% Cu-doped Cs_2ZnCl_4 NC sample with a magnification of the 380-420 nm region (inset).

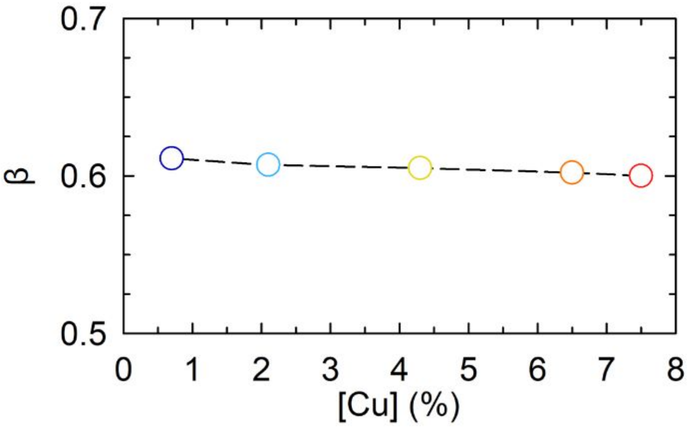

Figure S10. Stretching factor, β, of Cu-doped Cs_2ZnCl_4 NCs obtained by the fitting procedure of the PL decay curves with a stretched exponential function as reported in Figure 2c.

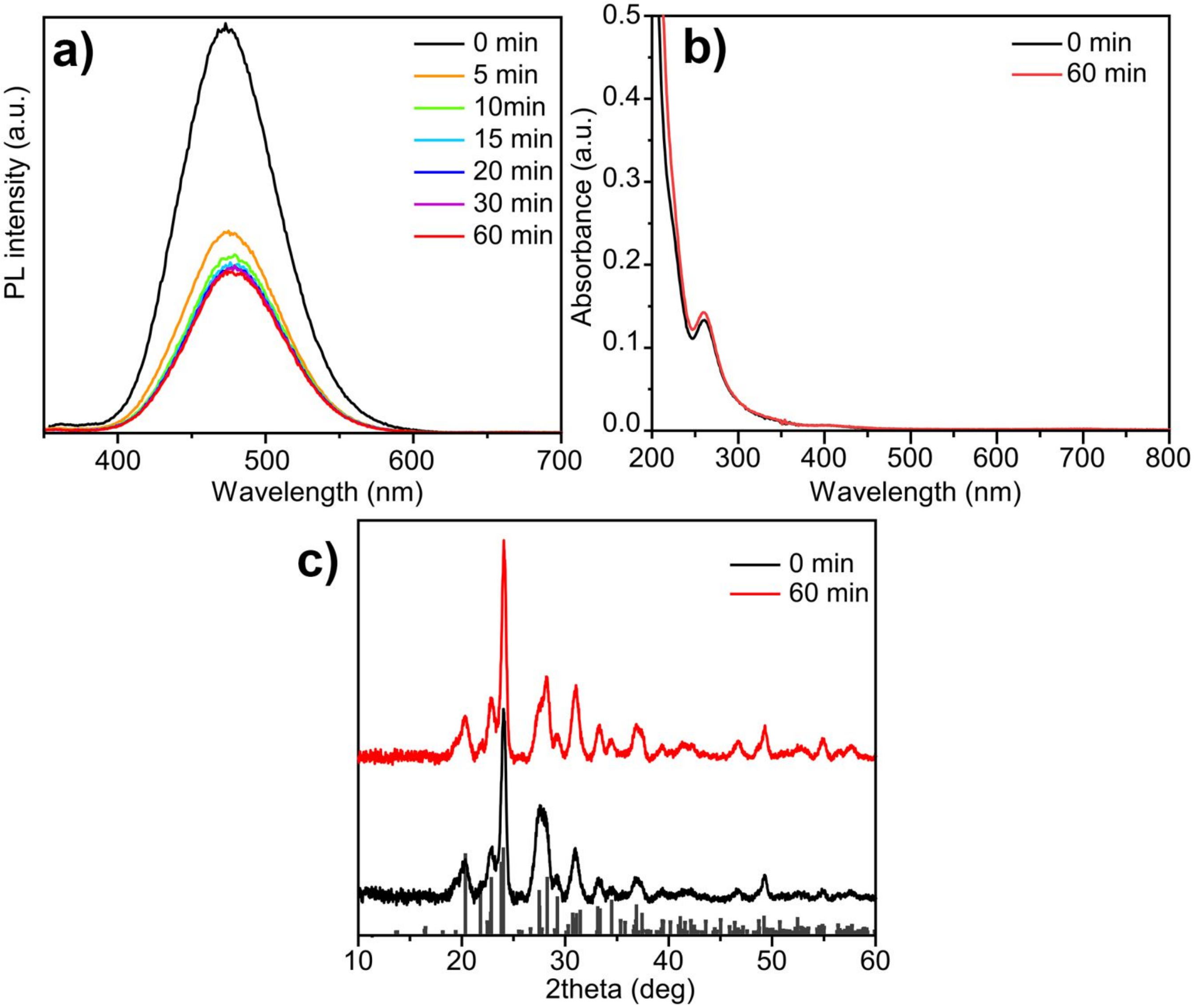

Figure S11. a) PL and b) absorption spectra of 2.1% Cu-doped Cs_2ZnCl_4 NCs dispersed in hexane and exposed to air up to 60min. c) XRD patterns of 2.1% Cu-doped Cs2ZnCl4 NCs before and after 60 min of exposure to air.

Table S2. Bond and angle parameters of metal chloride units embedded in the 0D structure computed at the DFT/PBE level of theory on a 2x2x2 cell and at the Γ point. In some cases, the copper chloride moieties present geometrical parameters that slightly deviate from the ideal symmetry due to a different interaction with the surrounding embedding media.

Structural Parameter [M = Zn, Cu]	Cs_2ZnCl_4	Cu(II):Cs_2ZnCl_4 with Cu(II) as $[CuCl_4]^{3-}$ unit	Cu(I):Cs_2ZnCl_4 (absorption) with Cu(I) as $[CuCl_3]^{2-}$ unit	Cu(I):Cs_2ZnCl_4 (emissive state) with Cu(I) as $[CuCl_3]^{2-}$ unit
M-Cl (Å)	~2.29	2.26/2.28/2.28/2.28	2.23/2.26/2.29	2.28/2.39/2.41
Cl-M-Cl (deg)	~109	98/102/122/132	113/118/129	95/100/113
Cl-M-Cl-Cl (deg)	~120 (tetrahedral)	~115 (pseudo-tetrahedral)	~3-5 (trigonal planar)	~115 (trigonal pyramidal)

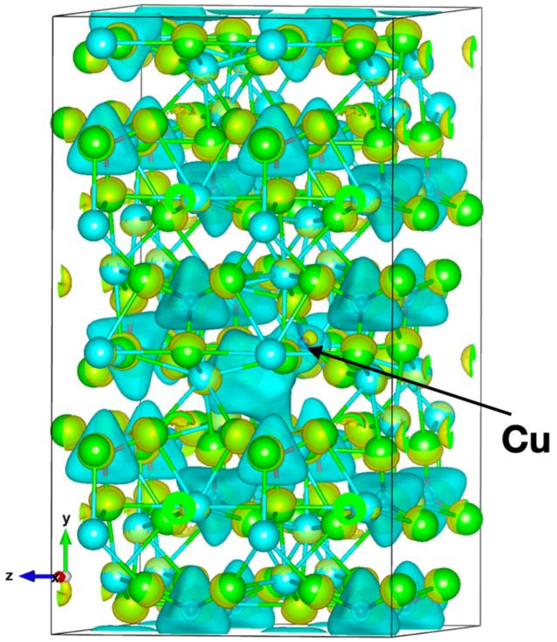

Figure S12. Molecular orbital plot of the lowest state of the conduction band, referred to the Figure 3e in the main text. An interesting feature here is that conduction band states are delocalized and composed by the linear combination of empty 4s orbitals of Cu with both the 6s of Cs and 4s of Zn. Color-code: Cs(cyan), Cu(blue), Zn(red), Cl(green).

Synthesis of undoped and Cu-doped Cs_2ZnBr_4 and Cs_2ZnI_4 NCs

We employed the same reaction strategy reported in the main text and we substituted benzoyl chloride with either benzoyl bromide or iodide to produce also Cu-doped Cs_2ZnBr_4 and Cs_2ZnI_4 NCs. We could not obtain Cs_2ZnI_4 nor Cu-doped Cs_2ZnI_4 NCs, while in the case of both undoped and Cu-doped Cs_2ZnBr_4 NCs we observed the formation of elongated parallelepiped NCs (rod-like) (Figure S13 c-d) exhibiting the orthorhombic Cs_2ZnBr_4 crystal structure (ICSD number 41537) (Figure S13 a). The synthesis was carried out at 150°C and the reaction time was 0s, that is, the reaction was quenched immediately after the injection of Bz-Br. While undoped Cs_2ZnBr_4 did not exibith any PL emission, Cu-doped NCs were characterized by a PL peak located at 465 nm with the corresponding PLE peak located at 270 nm (Figure S13 b).

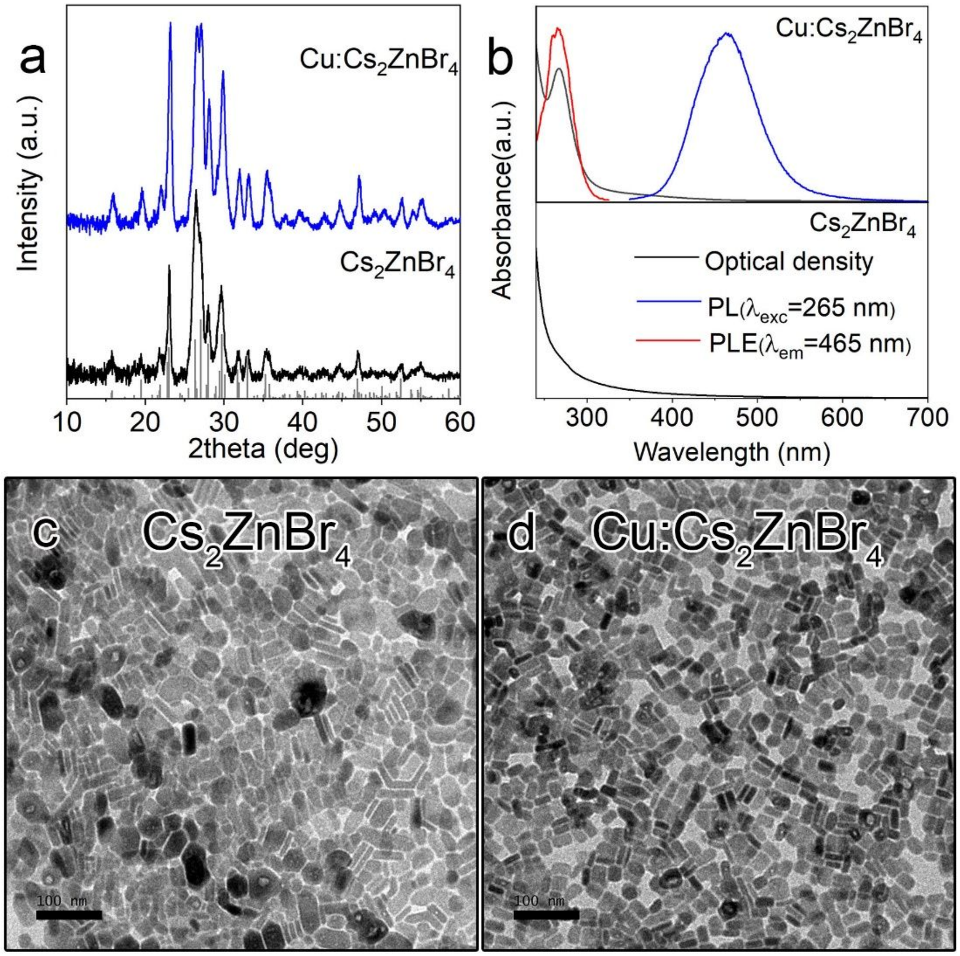

Figure S13. a) XRD patterns, b) optical properties and c-d) TEM micrographs of undoped and Cu-doped Cs_2ZnBr_4 NCs
